# The Hospital World

**Published:** 1910-02-12

**Authors:** 


### The Hospital World.

### Hospital Bureau of Standards and Supplies.

Representatives of the larger hospitals of New York City met at the Roosevelt Hospital recently and organised a Hospital Bureau of Standards and Supplies. This bureau will immediately establish a purchasing agency in charge of a trained buyer. It is estimated by Mr. Thorne, treasurer of the Presbyterian Hospital, who organised the purchasing departments of the Union and Southern Pacific railroads, that a saving of from 10 to 20 per cent. will be effected in the purchase of hospital supplies, through the establishment of this central buying station.

### The Manchester and District Association of Hospital Secretaries.

This Association was founded on March 30, 1908, and has a large and increasing membership, restricted to hospital secretaries engaged in the administration of voluntary hospitals within fifty miles of Manchester. The Association numbers as its members secretaries of voluntary hospitals in Liverpool, Manchester, Oldham, Preston, Bolton, Blackburn, Bury, Southport, Stockport, Buxton, Macclesfield, Ashton-under-Lyne, and Altrincham. The Association is private, and holds four quarterly meetings every year, when visits are made to voluntary hospitals and papers on hospital management are read and discussed. Though barely two years have passed since its foundation, seven hospitals have been visited: the Crossley Sanatorium, Delamere Forest; the old and new Manchester Royal Infirmary; Hospital for Incurables, Heaton Mersey, Cheshire; St. Mary's Hospitals, Manchester; and the Oldham Infirmary; and this year a visit will be paid to Liverpool, and the Northern and the Royal Southern Hospitals. It is satisfactory to note that not only has the Association a large membership, but it has also the support of the Chairmen and Boards of Management of the voluntary hospitals in the North. In many cases its members have been welcomed not only by representatives of the managing body, but also of the honorary staffs, and this intercourse has been of great value to the hospital secretaries on these enjoyable visits. The social side has not been neglected and the annual dinner is a feature of its activity, inasmuch as it enables the members to gather once a year and throw aside "Hospitalism" for once. The President is Mr. Walter G. Carnt, F.C.I.S., General Superintendent of the Manchester Royal Infirmary, and Mr. H. J. Eason, Secretary of the Manchester Children's Hospital, is the Hon. Secretary and Treasurer.

### Consumption Shelters.

A sub-committee of the Romford Guardians recently inspected Dr. Lyster's sanatorium at Little Baddow, Essex, which was started ten years ago. After several experiments a canvas shelter was constructed which could be opened and closed at will. By this means the patients get a maximum of light and air, as air percolates through the canvas when the shelter is entirely closed. Shelters were erected for the inhabitants, by subscription, some being placed in the cottagers' gardens, and at the present time tuberculosis has been stamped out in the villages of Great Baddow, Little Baddow, and Danbury. Some of the patients have lived in the shelters for five years. All the cases in the district now are imported ones. The Sanatorium at Little Baddow consists of a day-room for the patients, and five shelters at present. Other shelters are in course of erection. They are called the "Alfred Boyd Memorial Homes for Treatment of Consumption." A tablet states "These shelters were erected and furnished in loving memory of the late Alfred Boyd, of Great Gibcracks, Sandon, Essex, 1909." The shelters are built with wood frames, and have canvas curtains on four sides, with canvas roof. The bottom curtains are of medium flax canvas, the top curtains light lax canvas, and the roof of dressing canvas. They are built to accommodate two patients in each, the dimensions being 12 feet square, 8 feet to apex of roof, and 6 feet to eaves. The cost of each shelter is £12. The initial cost per patient works out at £16 each, or £32 per shelter, which includes beds, tables, cupboards, lamps, etc. The Committee strongly recommends the erection of such shelters, and also the establishment of a consumption sanatorium on the butterfly system.

### Hospital Advertising.

The Sphinx Club, the members of which are interested in all branches of advertising, dined at the Hôtel Cecil on February 2, Mr. Ralston Balch being in the chair. Mr. Balch opened the discussion by saying that it was idle to suppose that a charity which did not have good advertising could have a good revenue. Mr. Sydney Holland then related some of his experiences at the Poplar and London Hospitals. He said that if advertisement was to be of any use it must be original. One way might be "by calling a lady names," but "the best way of all to help a hospital was by a letter to the newspapers, only it must be written at the right moment and end with a sob or a smile." "It was useless to send a letter signed by three millionaires, a Bishop, and a society lady." "I used to acknowledge subscriptions in the agony columns, adding 'six accidents an hour.' That was a useful line." "Then I used to advertise that I was never in debt, and never intended to be. That was the only advertisement that ever paid." "Englishmen will always help a hospital that is not in debt. People are always ready to help those who are not in debt." "The main use of advertisements was not to bring in money, but, like John the Baptist, to pave the way for letters which had more chance of insertion if they came from a good customer." Sir John Kirk, who followed, said that appeals for boots and clothing did not attract people like appeals for funds to feed them. It was a great advantage for an individual to remain a long time with a society; people seemed to cluster round an individual rather than an abstract committee.

### Personalia.

Mr. Joseph S. Barber having resigned the post of Senior Dispenser to the Royal Free Hospital, which he has held for the remarkably long period of 59 years, the Committee of Management has granted him a substantial pension in recognition of his long and faithful service. The members of the committee, and medical and nursing staff of the hospital have presented Mr. Barber with a clock and easy chair as a testimonial of their esteem.

Mr. William Macadie, Ph.C., of the Hospital for Sick Children, Great Ormond Street, W.C., has been appointed senior dispenser at the Royal Free Hospital, Gray's Inn Road, W.C., in succession to Mr. Barber. The appointment was made by the General Committee of the hospital, assisted by the Drug Committee (Mr. C. B. Allen, Mr. H. J. Kluge, and Dr. W. H. Martindale). Mr. Macadie was formerly an assistant in retail pharmacies in Clifton, Cardiff, and Swansea. In 1904 he passed the Major from the London College of Chemistry and Pharmacy, and has been chief assistant dispenser at Great Ormond Street for five years.

